# Exploiting induced pluripotent stem cell-derived retinal pigment epithelium to unravel host-pathogen interaction in ocular tuberculosis: a reverse translational *in vitro* model

**DOI:** 10.3389/fopht.2025.1610215

**Published:** 2025-06-04

**Authors:** Ikhwanuliman Putera, Sanne van de Meerendonk, Nicole M.A. Nagtzaam, Rina La Distia Nora, Saskia M. Rombach, Jurriaan E.M. de Steenwinkel, Johannes R. Vingerling, Willem A. Dik, P. Martin van Hagen

**Affiliations:** ^1^ Department of Ophthalmology, Faculty of Medicine, University of Indonesia – Cipto Mangunkusumo Hospital, Jakarta, Indonesia; ^2^ Department of Ophthalmology, Erasmus University Medical Center, Rotterdam, Netherlands; ^3^ Department of Internal Medicine Section Allergy & Clinical Immunology, Erasmus University Medical Center, Rotterdam, Netherlands; ^4^ Laboratory Medical Immunology, Department of Immunology, Erasmus University Medical Center, Rotterdam, Netherlands; ^5^ Department of Medical Microbiology & Infectious Diseases, Erasmus University Medical Center, Rotterdam, Netherlands; ^6^ Laboratory Medical Immunology, Reinier Haga Medisch Diagnostisch Centrum (RHMDC), Delft, Netherlands; ^7^ Department of Immunology, Faculty of Medicine, Chulalongkorn University, Bangkok, Thailand; ^8^ Department of Internal Medicine, Faculty of Medicine, University of Indonesia – Cipto Mangunkusumo Hospital, Jakarta, Indonesia

**Keywords:** induced pluripotent stem cells, personalized medicine, retinal pigment epithelium, uveitis, tuberculosis

## Abstract

*Mycobacterium tuberculosis* (*Mtb*) can infect the retinal pigment epithelium (RPE) cells. Current *in vitro* research models for ocular tuberculosis (OTB) only rely on RPE cell culture approaches. Until now it remains unclear why only a minority of patients with active systemic tuberculosis (TB) develops concurrent OTB. There is significant variation in the clinical manifestations of OTB, which is potentially influenced by ethnic differences and diversity in mycobacterial strains. To better understand the immunopathobiology of OTB, particularly an individual’s susceptibility to *Mtb*-infection and the specific host response, cell culture systems utilizing induced pluripotent stem cells (iPSC)-derived RPE cells offer a promising *in vitro* model to better mimic the disease. With this technology, RPE cells can be generated from specific patients of interest, enabling to test hypotheses in a bench to bedside or reverse manner. In this current study, we explore the utility of iPSC-derived RPE cells as an *in vitro* model for OTB. Such an approach would overcome drawbacks associated with the currently commonly used “general” RPE cell lines as disease model. The application of iPSC-derived RPE cells offers promising options for the identification of novel biomarkers and to study individualized drug screening methods for host-directed therapy of OTB, in order to restore and maintain vision in OTB patients with sight-threatening disease.

## 1 Introduction

Tuberculosis (TB) is a disease caused by *Mycobacterium tuberculosis* (*Mtb*) and affects an estimated 10.8 million people globally, according to the latest 2023 World Health Organization (WHO) report (1). However, only about 5-10% of individuals infected with *Mtb* will develop active TB, while most remain in a latent or non-active disease state (2). Approximately 10% of those with latent TB will develop active disease at some point in their lifetime (2). Interestingly, ocular TB (OTB) is reported to affect only 1–7% of patients with active systemic TB (3–6). However, from an ophthalmologist’s perspective, uveitis due to active systemic TB accounts for about 8% of all uveitis cases in high TB-burden countries such as Indonesia and India, making it the second leading cause of infectious uveitis (7). In clinical practice, OTB has diverse clinical phenotypes (8). Although choroidal granuloma is considered as a hallmark manifestation, it is observed in less than 20% of uveitis cases associated with active systemic TB (9). Other choroidal lesions, such as TB-serpiginous-like choroiditis (TB-SLC) and multifocal choroiditis, can also be present in varying proportions (9). Interestingly, most reported cases of TB-SLC originate from India (10), where this phenomenon is rarely seen in Indonesia (11), despite the high TB incidence in both India and Indonesia (1). This raises several important questions:

1. Why do only a subset of patients with active systemic TB develop OTB?
2. What underlying immunopathobiological processes contribute to the significant variability in the clinical presentation of OTB, especially given the differences observed across ethnicities (e.g., Indian versus Indonesian population) despite similar TB burdens?
3. Do different *Mtb* strains play a role in susceptibility and clinical manifestations of OTB across different settings?

Current observational clinical studies offer valuable insights into the differences in the manifestations of OTB, but they are limited in exploring underlying disease mechanisms. To date, no study has specifically focused on the contribution of genetic background on OTB.

While *in vitro* disease models using human retinal pigment epithelial (RPE) cell lines can help clarify the host response to *Mtb* infection (12, 13), and animal models are better suited to assess disease pathogenesis (14, 15), these models still fall short when it comes to answering complex questions like those mentioned above. It is important to note that *Mtb* infections are likely influenced by host genetic susceptibility. This is exemplified by the pathobiological mechanism observed in Mendelian Susceptibility to Mycobacterial Diseases (MSMD), which highlights the crucial role of genes involved in interferon (IFN)-γ-IL-12 signaling (16, 17). The identification of this altered signaling pathway has led to studies that used IFN-γ therapy alongside antimycobacterial drugs, which proved effective in treating patients with MSMD (17). However, host genetic susceptibility to TB is multifaceted and likely involves multiple genes (2, 18).

The use of induced pluripotent stem cell (iPSC)-derived *in vitro* models offers a promising approach to better recapitulate disease processes, providing insights that are not achievable through traditional *in vitro* models using cell lines or animal models (19). One advantage of iPSC-derived RPE cells is their ability to study not only single-gene diseases but also complex diseases involving multiple genes and pathways, as the reprogrammed iPSC retain the genetic component of the donor (19). Beyond that, this article explores the application of the reverse translational research paradigm, by addressing clinical questions and testing hypotheses through a bedside-to-benchtop approach (20–22). This paradigm holds promise for identifying novel biomarkers and developing personalized therapeutic strategies for OTB—an area that remains underexplored so far. Current treatment strategies for OTB follow “group” protocols designed for pulmonary or extrapulmonary TB in general (23). If the different phenotypic manifestations of OTB result from distinct underlying mechanisms, understanding the variations in the host immune response across clinical phenotypes and ethnic backgrounds could provide a basis for identifying compounds that may serve as more tailored therapies.

## 2 Current *in vitro* disease model of ocular tuberculosis

Since the observation that *Mtb* resided in the RPE layer of cadaveric eyes with OTB (24), RPE cell lines have been utilized to study host-pathogen interactions in OTB (12, 13, 25). The ARPE-19 is an RPE cell line derived from the eyes of a healthy 19-year-old male who passed away due to a traffic accident and has been extensively used for *in vitro* studies on RPE characterization (26), and as a model for ocular TB infection (25). Additionally, primary RPE cell lines, derived from donor eyes have also been described (27). Using such a primary cell line, our group has characterized the cellular response of RPE cells to *Mtb* infection and demonstrated that RPE cells are permissible to *Mtb* infection and are capable of eliciting a defense response that resembles that of *Mtb*-infected macrophages, albeit with lower magnitude (12). Importantly, we also identified that RPE respond with strong activation of IFN signaling, primarily IFN type 1 in response to *Mtb* infection (12). Although these studies provide important insight into the immunopathobiology of OTB, the clinical heterogeneity of OTB cannot be fully recapitulated and explained by these models. It remains unclear whether the *Mtb*-elicited immune response we observed in these primary RPE cells reflects a general RPE-response or whether RPE cells from individuals with different genetic make-up can have different susceptibility to *Mtb* infection with subsequent differences in immune activation. If such differences in response would exist between individuals we hypothesize that this could contribute to clinical heterogeneity, and potentially also the response to treatment, including host directed therapies (28). This highlights the need for a more innovative and creative approach for better *in vitro* models to study the ocular response to *Mtb* against different genetic back-grounds.

## 3 Application of induced pluripotent stem-cell-derived retinal pigment epithelium to study ocular tuberculosis: prospects and challenges

In this study, we present the use of iPSC technology to generate RPE cells. Application of this technology would enable the study of the RPE response to *Mtb* on a per individual basis. As a proof of principle we here use an already established iPSC clone that was derived from a donor without evidence of systemic or ocular TB. From this iPSC we generated RPE that were subsequently infected with *Mtb*, as we described previously (12) and examined the response to infection by these iPSC-derived RPE cells.

### 3.1 Generation of iPSC-derived RPE cells

**Figure 1:**
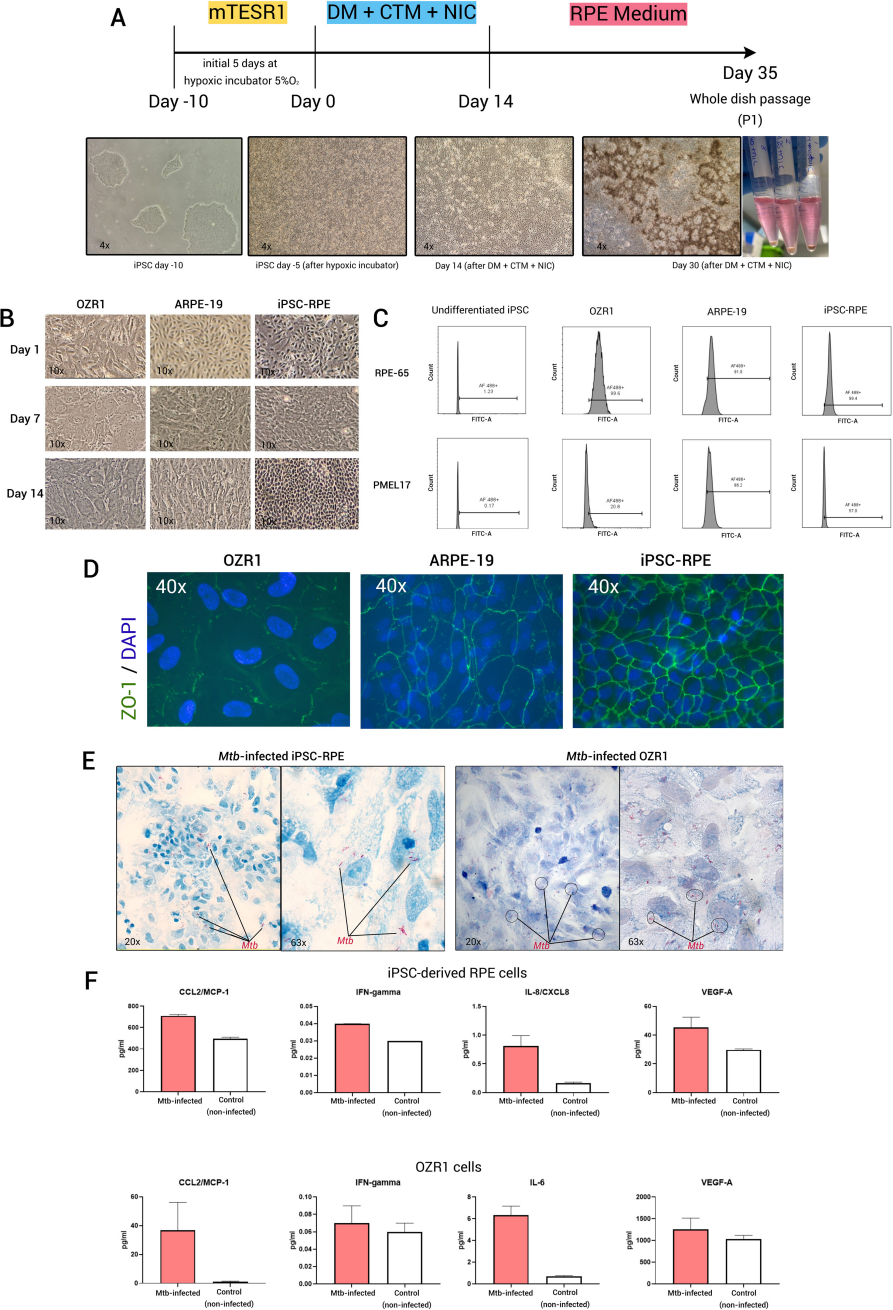
Generation of iPSC-derived RPE and its utilization as *in vitro* OTB model. **(A)** Schematic of iPSC-derived RPE generation using chetomin (CTM) and nicotinamide (NIC) treatment during the differentiation protocol, **(B)** Bright field microscopy images (10x magnification) of OZR1, ARPE-19, and iPSC-derived RPE cells at different time points, **(C)** Flow-cytometric analysis of the expression of RPE markers (RPE-65 and PMEL17) in iPSC-derived RPE compared to OZR1 cells, ARPE-19 cells, and undifferentiated iPSCs. iPSC-derived RPE cells (passage 2), ARPE-19 cells, and OZR1 cells were cultured for 21 days. Fraction (%) of viable cells within the sample for each marker were presented. Thresholds were determined from unstained controls. Data were generated from a single experiment, **(D)** fluorescence microscope images with similar 40x magnification in all slides of iPSC-derived RPE (passage 2), ARPE-19, and OZR1 cells immunostained for ZO-1 (t = 21 days), **(E)** Microscopy image of iPSC-derived RPE and OZR1 cells at 48 hours after infection with live H37Rv *Mtb* (AFB Kinyoun staining: 20x magnification (left) and 63x magnification (right)), **(F)** Bar graphs represent the level of proteins measured in the culture supernatants of *Mtb*-infected (strain H37Rv, red bars) versus non-infected controls (white bars) iPSC-derived RPE and OZR1 cells (t = 48 hours). Data were generated from two independent experiments.

The human iPSC clone was obtained from the iPS Core Facility at Erasmus MC, the Netherlands. The cells (EMC229i, clone 20) were sourced from a 25-year-old healthy male of Caucasian (Dutch) origin. Various methods for generating RPE cells from iPSCs have been previously summarized (29, 30). In this study we used a validated protocol described by Maruotti et al. (31) (see **Figure 1A** for a schematic flow of the differentiation process and **Supplementary Information**). When comparing the morphological appearance of iPSC-derived RPE cells to OZR1 and ARPE-19 cells under direct bright microscopy, we observed that the iPSC-derived RPE cells reached an almost uniformly hexagonal shape more rapidly than OZR1 and ARPE-19 (**Figure 1B**). We confirmed that the differentiation protocol effectively induced the expression of genes associated with RPE differentiation (*OTX2*, *MITF*, and *PMEL17*), consistent with the protocol by Maruotti et al. (31). As we followed a previously established protocol, real-time polymerase chain reaction (RT-PCR) to assess early differentiation markers was performed in duplicate from a single experiment (**Supplementary File**). The high expression of the three markers (*OTX2, MITF*, and *PMEL17*) in iPSCs treated with differentiation medium (DM) in combination with chetomin (CTM) and nicotinamide (NIC), compared to undifferentiated iPSCs or iPSCs treated with DM without CTM, indicates successful differentiation toward RPE at Week 2 (as outlined in the differentiation scheme in **Figure 1A**), prior to the maturation phase initiated by switching to RPE medium. The generated RPE cells exhibited typical morphological features and pigmentation characteristics under direct microscopy by days 30–40 of differentiation.

After the first cell passaging, flow cytometric analysis of key mature RPE markers (RPE-65 and PMEL17) demonstrated expression levels in the iPSC-derived RPE that were comparable to those observed in OZR1 and ARPE-19 (**Figure 1C**). In addition, the iPSC-derived RPE cells we generated also expressed more pronounced membrane expression of the tight junction molecule ZO-1, more closely resembling the well-organized morphology of RPE cells than the currently established RPE cell lines (OZR1 and ARPE-19) at the same time point (**Figure 1D**). iPSC-derived RPE proliferated more rapidly with a hexagonal shape and a more tightly packed arrangement compared to the other two cell lines. The functionality of the iPSC-derived RPE (e.g., polarity and phagocytic capacity) was previously described in the sourced protocol and were not repeated in the current study.

### 3.2 Internalization of *Mtb* by iPSC-derived RPE cells

In our previous work, we demonstrated that both OZR-1 and ARPE-19 cells can be infected by *Mtb* (12). We observed relatively similar host response profiles at the protein secretion level following infection in both cell lines (12). Based on these findings, we proceeded with pathway analysis using OZR-1 cells, as no substantial differences were anticipated between the two. In the current study, following the characterization of mature RPE cells across all three cell types (**Figures 1C, D**), OZR-1 was selected for comparison with iPSC-derived RPE cells to demonstrate that the iPSC-derived RPE can also be infected by *Mtb*. The iPSC-derived RPE were infected with *Mtb*, as previously described (Multiplicity of infection (MOI) 1:10) (12). Microscopic examination was conducted after 48 hours of infection. Acid-fast bacilli (AFB) Kinyoun stained slides revealed that iPSC-derived RPE cells internalized the *Mtb*, similar to OZR1 cells (**Figure 1E**). For measurement of culture supernatant cytokines, two culture supernatant samples per experimental group were taken out of the *Mtb* biosafety level 3 laboratory for analysis. Measurements of a set of 12 pre-selected cytokines/chemokines (see **Supplementary Material**) in the culture supernatants revealed secretions patterns that where comparable between iPSC-derived RPE cells and OZR1 cells. CCL2, IFN-γ, IL-8/CXCL8, and VEGF-A were elevated in culture supernatants from *Mtb-*infected iPSC-derived RPE cells. In case of *Mtb-*infected OZR1 cells CCL2, IFN-γ, IL-6, and VEGF-A were elevated in culture supernatants (**Figure 1F**). The remaining cytokines were undetectable.

### 3.3 Potential utility of in vitro OTB model using iPSC-derived RPE cells

**Figure 2:**
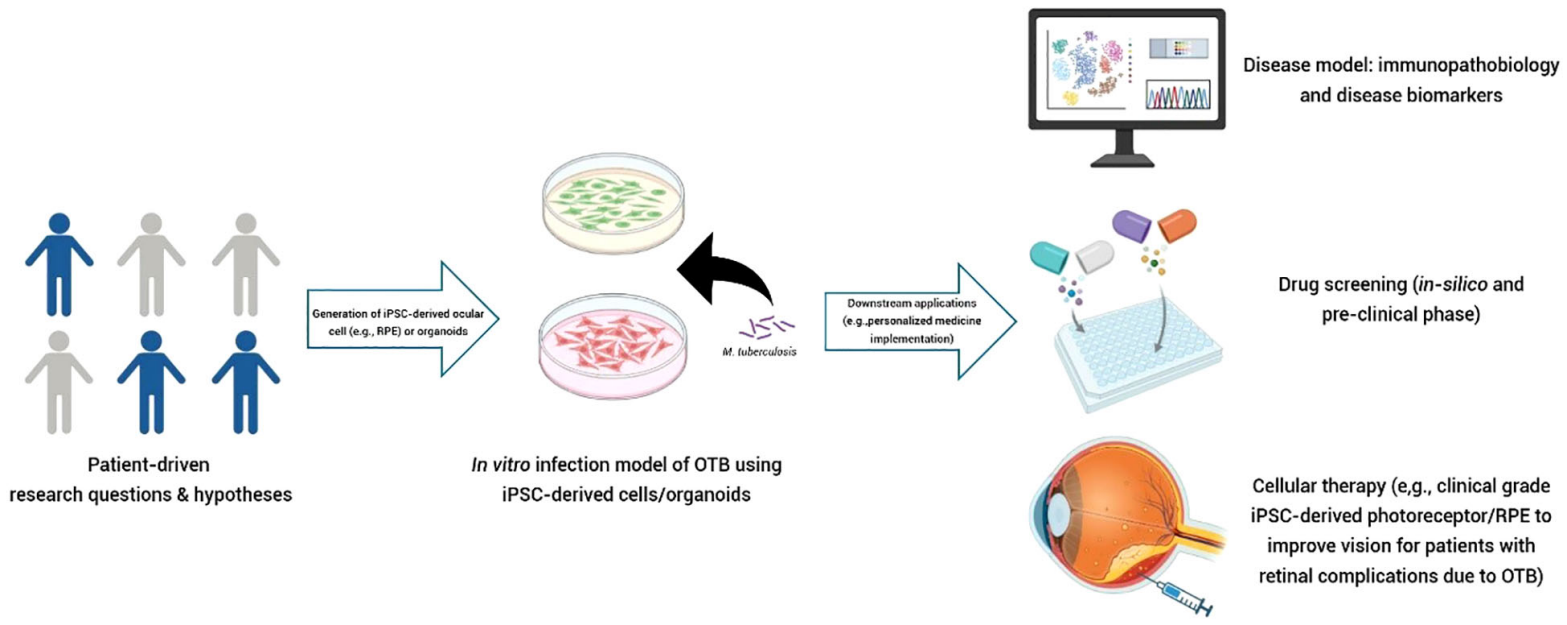
Potential application of iPSC-derived cells (e.g., RPE) as a reverse translational *in vitro* model in OTB. Testable hypotheses arising from questions in clinical care can be explored using this *in vitro* concept. iPSC-derived cells can be sourced from patients/individuals with diverse characteristics, such as different clinical phenotypes, ethnic backgrounds, or disease manifestations (e.g., active pulmonary TB with and without OTB). These cells can then be expanded to create a “village on a dish”. High-throughput analysis can be employed to characterize the disease, identify novel biomarkers, or discover therapeutic targets. This technology can be utilized to perform “clinical trials in a dish.” Clinical-grade of patient-derived cells (autologous or allogeneic) also hold promise as candidates for cellular therapy in restoring damaged ocular structure due to OTB. (Figure created with Biorender.com).

Research on iPSC-derived RPE cells has so far primarily focused on age-related macular degeneration (AMD) and inherited retinal diseases, with no studies yet that investigated their potential for delineating the contribution of RPE to (infectious) uveitis. A recent study by Voisin et al. explored the mechanisms behind the distinct phenotypes of AMD: atrophic and exudative (32). The study found that variations in the expression patterns of three genes (*ABCA1, RB1CC1*, and *RPN2* - all involved in the waste clearance pathway of RPE) likely contribute to the different disease phenotypes (32). It is important to emphasize that a particular disease requires an appropriate *in vitro* system as a reliable disease model. Cai et al. previously compared *in vitro* models of AMD, using fibroblasts and iPSC-derived RPE cells from AMD patients (33). Their findings showed that iPSC-derived RPE cells more accurately recapitulated the disease than fibroblasts (33). Due to the difficulty of directly obtaining eye-derived cells, the development iPSC-derived cell technology now allows for the generation of ocular cells without the need to directly harvest ocular tissue from patients. This advancement is also likely to be crucial for the discovery of new treatment strategies for OTB. An intriguing question remains why only a minority of patients with active systemic TB develop concurrent OTB. Could it be that these OTB patients have RPE cells that are more prone to harboring disseminated *Mtb*? If specific signaling pathways are altered in these patients and can be identified, it could potentially lead to the discovery of novel biomarkers and adjunctive treatments to better combat *Mtb* infection in their eyes. We expect that by utilizing iPSC-derived RPE cells from a wide range of sources, including individuals from different ethnic backgrounds and those with varying manifestations of OTB, we may be able to uncover mechanisms of individual susceptibility to OTB. This approach could also shed light on the different clinical presentations of the disease (**Figure 2**).

TB treatment involves a combination of antitubercular drugs typically administered for a minimum of six months (23). However, in cases of OTB, there is some debate on whether a six-month regimen is sufficient, or if treatment should be extended to at least nine months, as recommended for extrapulmonary TB (23). Importantly, immunosuppressants, such as systemic corticosteroids, are often prescribed to help resolve the intraocular inflammation (23). The final visual outcome of OTB largely depends on the successful elimination of *Mtb*, effective reduction of intraocular inflammation to minimize structural damage to ocular tissues, and the prevention of complications like macular edema. Since uveitis resolution in OTB can take several months (34), identifying effective adjunctive treatments that can reduce local inflammation while also facilitating *Mtb* elimination alongside standard ATT remains a significant area of research (28). This fits with the concept of host-directed therapy for TB management (28). It is important to mention that the development of new anti-infective TB drugs faces significant challenges, including the lack of reliable and scalable models for organ-specific drug screening (35). Additionally, the drug discovery process can take 10–15 years with costs over $1–2 billion to implement a new therapy into clinical care (36). Remarkably, about 9 out of 10 drug candidates entering phase 1 trials ultimately fail, with up to 50% of these failures due to efficacy issues (36). One key factor contributing to these failures is thought to be the discrepancy between the disease models used in drug screening and the actual disease pathology (37).

The emergence of iPSC-derived cell technology offers a promising alternative, providing potentially superior tools for *in vitro* disease modeling in comparison to currently used models (38). These advancements could, in theory, enhance the relevance of preclinical models, improve drug screening accuracy, and aid in the identification of more effective treatments (38–40). A recent study by Sequeira et al. demonstrated the successful identification of effective drugs for a patient with Leigh-like syndrome, a rare progressive mitochondrial disorders of oxidative phosphorylation (41). The patient had previously participated in clinical trials with unfavorable results (41). By generating patient-specific iPSC-derived fibroblasts, neural progenitor cells, and cardiomyocytes, the researches created a personalized drug screening platform that ultimately enabled them to identify drugs that were effective for the patient (41).

Similar research approaches on TB are expected to gain significant momentum in the near future. A study by Han et al. highlighted significant differences in the transcriptomic profiles and cellular responses to *Mtb* infection among commonly used macrophage models, including THP-1 cells (derived from a monocytic leukemia cell line), human monocyte-derived macrophages (hMDMs), and iPSC-derived macrophages (iMACs) (42). Their study using iMACs successfully identified 10-DEBC hydrochloride (10-DEBC) as a potential therapeutic agent against *Mtb (* 42). In a separate study involving iMACs, 10-DEBC was also found to be effective against *M. abscessus (* 43). It remains to be seen whether this drug will eventually proceed into human trials and prove its efficacy. Given the potential of this technology for specific TB manifestations, such as OTB, applying this approach to identify novel adjunctive treatments for OTB is a promising and warranted direction for future research.

While exploring the potential of iPSC-derived RPE as an OTB disease model to better recapitulate the disease, it is important to note that the current generation of iPSC-derived RPE is still labor-intensive and costly. Recent studies have shown that while iPSC-derived RPE are morphologically and functionally similar to mature RPE, it still expresses genes related to chromatin regulation, indicating some degree of immaturity (44). Furthermore, extended cell culture periods improve cell maturity, making them more suitable for disease modeling (45). Therefore, efforts to simplify the procedure and shorten the generation time of iPSC-derived RPE, while preserving their maturity, are still required. Further research utilizing this technology in developing countries should also be encouraged to expand its applications and accelerate scientific progress. Moreover, further advancements are necessary to create clinical-grade iPSC-derived RPE that not only recapitulate mature RPE but which are also safe for patient treatment with effective improvement in vision.

## 4 Conclusion

The recent advancements in generating iPSC-derived RPE cells provide a powerful tool for better *in vitro* replication of human ocular diseases, including OTB. This technology offers a valuable model for gaining deeper insights into the immunopathobiology of patient-specific clinical manifestations. It represents a crucial initial step in reverse translational research, helping to understand the diverse clinical phenotypes and susceptibilities to OTB, to identify novel biomarkers, and facilitate drug screening. While our current work utilized iPSC-derived RPE cells from an otherwise healthy donor, future research could replicate this approach using patient-specific sources to generate corresponding iPSC-derived RPE cells. This would allow for a more thorough analysis of host-pathogen interactions in OTB or other forms of uveitis. Furthermore, scaling up iPSC-derived RPE cells to clinical-grade applications holds promise as a potential cellular therapy for patients with late-stage disease, particularly those with retinal damage that cannot be cured with the current approaches.

## Data Availability

The raw data supporting the conclusions of this article will be made available by the authors, without undue reservation.
