# Supplementary material for: Exploiting induced pluripotent stem cell-derived retinal pigment epithelium to unravel host-pathogen interaction in ocular tuberculosis: a reverse translational in vitro model
Source: Front Ophthalmol (Lausanne). 2025 Jun 4;5:1610215. doi: 10.3389/fopht.2025.1610215 (PMC12173865; doi:10.3389/fopht.2025.1610215)
Supplement: Supplementary file 1 [file DataSheet1.docx]

**Supplementary Information**

*Generation of iPSC-derived RPE cells*

The human iPSC line used for RPE cell generation was obtained from the iPS Core Facility at Erasmus MC, the Netherlands. The cells (EMC229i, clone 20) were derived from a 25-year-old healthy male of Caucasian (Dutch) origin (from peripheral blood mononuclear cells). General informed consent for generating iPSCs from patient-derived materials to be used for research purposes has been obtained and is regulated by the iPS Core Facility at Erasmus MC, in accordance with the regulation of the local ethical committee (Erasmus MC). Reprogramming and quality checks of the iPSCs was performed by the iPS Core Facility using a standardized protocol with Sendai virus. Differentiation of the iPSC into RPE was performed following the published method by Maruotti *et al.^31^* (see Figure 1 for a schematic differentiation process), without any major modifications. The details of the differentiation protocol:

1. Standard iPSC culture (EMC229i, clone 20, passage 30) was performed with Matrigel® (Corning® Matrigel®, hESC-Qualified Matrix, Merck, CLS354277-1EA)-coated 6-well plates. The mTESR™1 medium (Stem Cell Technologies, # 85850) was refreshed every day until the day of RPE differentiation was initiated. The iPSC used for RPE differentiation were checked for mycoplasma test result was negative.
2. The iPSC colonies were seeded at high density (20,000 cells per cm^2^) on Matrigel®-coated plates and placed the cells in mTesR1 medium in hypoxic incubator (5% O_2,_ 5% CO_2_) for 5 days The culture medium mTESR™1 was refreshed after 48 hours in a hypoxic incubator.
3. After day 5 days, the cells were transferred to a normal incubator (5% CO_2_, 20% O_2_, 37ºC). The culture medium mTESR™1 was refreshed everyday (3 mL for each well). The cells appeared as a monolayer after 2 days.
4. After 10 days (step 1-3), the iPSC culture medium was replaced by differentiation medium (DM). The DM medium was prepared from a mixture of 425 ml DMEM/F-12, HEPES (Gibco™, 11574546) with an addition of 15% KnockOut™ Serum Replacement (Gibco™, 10828010), 2 mM L-Glutamine (Thermo Scientific, 25030032), 0.1 mM MEM Non-Essential Amino Acids Solution (Gibco™, 11140035), 1% Antibiotic-Antimycotic (Gibco™, 15240096), and 0.1 mM 2-Mercaptoethanol (Merck, M3148-25ml)
5. At the following day (Day 1), chetomin (CTM; Merck, C9623-1MG) and nicotinamide (NIC; Merck, N3376-100G) were introduced in addition to the DM. Three different concentrations of CTM were used: 10, 25, and 40 nM. NIC was used 10 mM concentration. The culture medium (DM) + CTM + NIC was refreshed every day until Day 14.
6. At Day 15, the culture medium was replaced by regular RPE culture medium. Just before medium switching, as we used a previously established protocol, real-time polymerase chain reaction (RT-PCR) to assess early differentiation markers was performed once, in duplicate, during this differentiation step (Supplementary Figure). The results showed that the three early RPE differentiation markers were expressed in iPSCs treated with varying doses of CTM, but not in undifferentiated iPSCs or iPSCs that were not treated with CTM. The RPE medium was prepared from a mixture of 70% DMEM (Invitrogen; 11965) with addition of 30% Ham's F-12 Nutrient Mix (Gibco™, 11765047), 2% B-27™ Supplement (Gibco™, 17504044), and 1% Antibiotic-Antimycotic (Gibco™, 15240096). The medium was refreshed every day.
7. At day 35, a whole dish passaging was performed. A new Matrigel®-coated 10-cm dish (BD) was prepared. The RPE medium was removed and 1 mL of Accumax™ solution (Merck, A7089-100ML) was added. The cells were incubated for 20 min at 37 °C, 5% CO_2_ until most cells appeared rounded and cells were vigorously dissociated using 1 ml pipet tip. RPE medium (2 ml) was added and the suspension was transferred into a 15-mL tube and centrifuged at 130 × g for 5 min. The supernatant was discarded and the cells were resuspended in RPE medium, the cell suspension was filtered through a 40-μm nylon mesh (BD Falcon). The cells were seeded at a density of 250–300,000 cells per cm^2^ in a new dish.


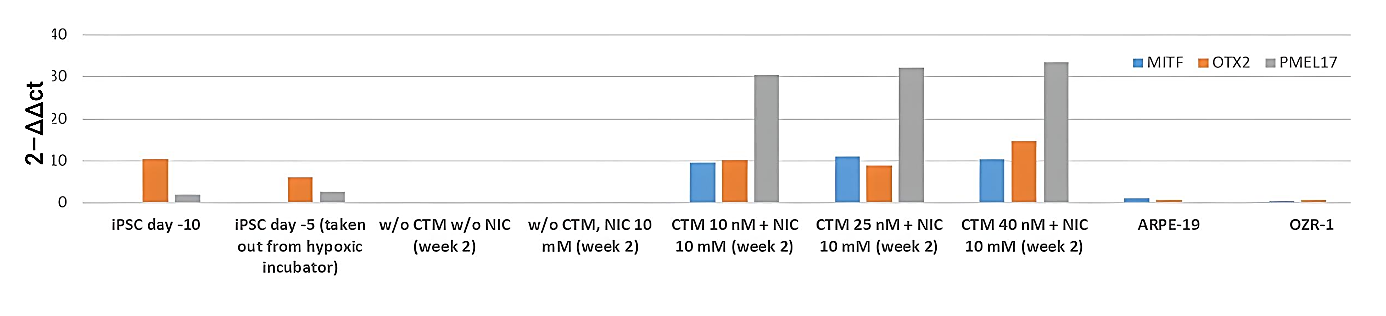


**Supplementary Figure.** Measurement of three selected RPE differentiation markers (*MITF, OTX2*, and *PMEL17*) using RT-PCR. PCR was conducted in duplicate from a single experiment.

*OZR1 and ARPE-19 cells culture*

OZR1 cells passage 9-12^th^ were cultured in Iscove's Modified Dulbecco's Medium (IMDM) supplemented with 10% fetal calf serum (FCS) and 1% penicillin-streptomycin. ARPE-19 cells (ATCC) passage 9-12^th^ were cultured in DMEM/F12 supplemented with 10% FCS and 1% penicillin-streptomycin. Culture medium for both OZR-1 and ARPE-19 cells was refreshed twice a week.

*Quantitative RT-PCR*

The iPSC-derived RPE cells displayed typical morphological features and pigmentation under direct microscopy by days 30–40. Successful differentiation into RPE was was further confirmed through RT-PCR analysis of selected genes (*MITF*; Hs01117294_m1; Thermo Fisher, 4453320; *OTX2*; Hs00222238_m1, Thermo Fisher, 4453320; and *PMEL17*; Hs00173854_m1, 4453320) during the differentiation process. Total RNA was isolated using RNeasy Mini Kit (Qiagen) according to the manufacturer’s instruction. The quality of the RNA was measured using NanoDrop ND1000 spectrophotometer (NanoDrop Technologies, USA). The relative expression levels of each gene of interest were normalized to the expression of the housekeeping gene *ABL* (2-ΔΔCt).

*Flow cytometry*

Immunostaining was performed using the PerFix expose kit (Beckman Coulter, B26976), according to the manufacturer’s instructions. iPSC-derived RPE cells at passage 2 were used for flow cytometric analysis. The expression of three RPE-associated markers, RPE-65 (Anti-RPE65 antibody [EPR22579-44], Abcam, ab231782) and PMEL17 (PMEL Monoclonal Antibody/OTI7E3, Thermo Fisher, TA500425), was determined. Goat anti-mouse and anti-rabbit conjugated to Alexa Fluor® 488 (Abcam) were used as secondary antibodies accordingly. Measurements were conducted on a Fortessa™ cytometer (BD Biosciences) and data were analysed with FlowJo software.

*Fluorescence microscopy*

Cells (OZR1, ARPE-19, and iPSC-derived RPE) were cultured on chamber slides and analysed for expression and distribution of the tight junction molecule zonula occludens-1 (ZO-1). For this, the cells were fixed with 4% paraformaldehyde for 10 minutes. Following washing twice with PBS, fixed cells were permeabilized with PBS containing 0.1% Triton-X for 10 minutes. Then, cells were washed again twice with PBS containing 0.05% Tween. Cells were incubated with PBS containing 1% fatty acid free bovine serum albumin (BSA) and 0.1% Tween 20 for 1 hour. Next, cells were stained with a Alexa Fluor488-conjugated ZO-1 antibody (1A12, Invitrogen, 339188) in 1% BSA/PBS 1:500 for 1 hour at room temperature. Cells were washed three times with PBS containing 0.1% Tween and then followed by DAPI staining^[[1]](#footnote-1)^. We used iPSC-derived RPE cells passage 2 that were previously differentiated using 40 nM CTM + 10 mM NIC. All images were taken from cells at day 21 after seeding.

*Mycobacterium tuberculosis* infection experiment

iPSC-derived RPE cells passage number 3 were resuspended in fresh culture medium (1.5 ml per well) without antibiotics one day prior to exposure with *Mtb* (strain H37Rv). In addition, OZR1 cells (passage 10) were also used and treated under similar conditions but were cultured under IMDM supplemented with 10% FCS and without using Matrigel®-coated chamber slides. Cells were maintained in a 5% CO_2_ incubator at 37 °C. For iPSC-derived RPE cells, cells were seeded overnight on Matrigel-precoated chamber slides (Nunc™ Lab-Tek™ Chamber Slide System, 177429PK; Thermo Fisher Scientific). Both iPSC-derived RPE and OZR1 were seeded at a density of 0.5 × 10⁶ cells/chamber.

The culture medium was refreshed before *Mtb* exposure. A pre-prepared *Mtb* suspension stored at −80°C was thawed at room temperature for 30 minutes, centrifuged (14,000 rpm, 10 minutes), and the supernatant was discarded. The pellet was re-suspended in phosphate-buffered saline (PBS). Cells were exposed to a multiplicity of infection (MOI) of 10 bacteria per cell (10:1) for 3 hours. Following a 3-hour incubation period, the medium was discarded, and cells were gently washed twice with 1 ml PBS. The cells were then incubated in the corresponding culture medium without antibiotics for 48 hours.

Non-*Mtb* exposed iPSC-derived RPE cells and OZR1 cells, otherwise cultured in the same manner, served as controls. Infection experiments were performed in a biosafety-level 3 (BSL-3) laboratory dedicated to tuberculosis research at Erasmus MC.

*Microscopy images of Acid Fast Bacili Kinyoun staining*

Cells seeded in chamber slides (0.5 x 10^6^ cells/chamber) were prepared for acid-fast bacilli (AFB) Kinyoun staining. After washing and air-drying, the chamber slide walls were removed, and the cells were fixed on a heated block at 100°C for 10 minutes prior to AFB Kinyoun staining. Images from the slides were acquired using a Carl Zeiss Axiocam 305 color microscopy camera.

*Measurements of proteins in culture supernatants*

Culture supernatants collected from chamber sides were analysed using a customized 12-plex human Luminex discovery assay (R&D system) for the following: CCL2, CCL5, CXCL9, CXCL10/IP-10, IFN-α, IFN-γ, IL-6, IL-8, IL-10, IL-12, TNF-α, and VEGF-A. The selection of these proteins was based on a previous study with *Mtb*-infected RPE cells (OZR1)^12^. Measurements of the supernatants were performed after passing the stored supernatant through an Eppendorf® membrane filter (EP4421601009) at a BSL-3 laboratory. Due to the limited number of available filters at the time of the experiments, only two culture supernatants per experimental group were taken out of the BSL-3 laboratory for Luminex analysis. The Luminex assay was further conducted according to the manufacturer’s instructions.

1. Bastiaans J, van Meurs JC, van Holten-Neelen C, Nagtzaam NM, van Hagen PM, Chambers RC, Hooijkaas H, Dik WA. Thrombin induces epithelial-mesenchymal transition and collagen production by retinal pigment epithelial cells via autocrine PDGF-receptor signaling. Invest Ophthalmol Vis Sci. 2013;54(13):8306-14. doi: 10.1167/iovs.13-12383. [↑](#footnote-ref-1)
